# Assessment of variability in traction interventions for patients with low back pain: a systematic review

**DOI:** 10.1186/s12998-018-0205-z

**Published:** 2018-09-17

**Authors:** Muhammad Alrwaily, Mohammed Almutiri, Michael Schneider

**Affiliations:** 1 Division of Physical Therapy, School of Medicine, West Virginia University, 1 Medical Center Drive, P.O. Box 9226 – Room 8304, Morgantown, WV 26506 USA; 2 King Fahad Specialist Hosptial, Dammam, Saudi Arabia; 3 Department of Physical Therapy, School of Applied Medical Sciences, Najran University, King Abdulaziz Rd, PO Box 1988, Najran, 61441 Saudi Arabia; 4 Department of Physical Therapy, School of Health and Rehabilitation Sciences, University of Pittsburgh, Bridgeside Point 1, 100 Technology Drive, Suite 210, Pittsburgh, PA 15219 USA

**Keywords:** Traction, Low back pain, Sciatica, Systematic review

## Abstract

**Background:**

Previous systematic reviews have concluded that lumbar traction is not effective for patients with low back pain (LBP), yet many clinicians continue to assert its clinical effectiveness.

**Objective:**

To systematically identify randomized controlled trials (RCTs) of traction and explore the variability of traction interventions used in each RCT.

**Method:**

A literature search started in September 2016 to retrieve systematic reviews and individual RCTs of lumbar traction. The term “lumbar traction” and other key words were used in the following databases: Cochrane Registry, MEDLINE, EMBASE, and CINAHL. The retrieved systematic reviews were used to extract individual RCTs. The most current systematic review included RCTs from inception until August 2012. We performed an additional literature search to update this systematic review with newer RCTs published between September 2012 and December 2016. All of the identified RCTs were combined and summarized into a single evidence table.

**Results:**

We identified a total of 37 traction RCTs that varied greatly in their method of traction intervention. The RCTs included several types of traction: mechanical (57%), auto-traction (16%), manual (10.8%), gravitational (8.1%) and aquatic (5.4%). There was also great variability in the types of traction force, rhythm, session duration and treatment frequency used in the RCTs. Patient characteristics were a mixture of acute, subacute and chronic LBP; with or without sciatica.

**Conclusion:**

There is wide variability in the type of traction, traction parameters and patient characteristics found among the RCTs of lumbar traction. The variability may call into question the conclusion that lumbar traction has little no or value on clinical outcomes. Also, this variability emphasizes the need for targeted delivery methods of traction that match appropriate dosages with specific subgroups of patients with LBP.

## Introduction

Lumbar traction is a commonly used method to treat patients with low back pain (LBP) with or without sciatica. In the UK and the US, lumbar traction is used by 41 and 77% of outpatient rehabilitation providers respectively [1, 2]. Despite this common use of lumbar traction in the clinical setting, several systematic reviews have concluded that lumbar traction has little or no value on the clinical outcomes of pain intensity and functional status. The reviews also suggest that traction does not appear to lead to quicker return to work among people with LBP with or without sciatica [3–5]. These conclusions present a clear discordance between evidence-based recommendations and how lumbar traction is regarded in current clinical practice [1, 2, 6].

The earliest systematic review, conducted in 1995, included 17 randomized controlled trials (RCTs) that assessed traction on neck and low back pain [7]. Of the 17 RCTs, only 3 (2 lumbar, 1 cervical) had good quality. This systematic review concluded that traction efficacy was unclear, and called for more proper design and better methodological quality in future traction trials.

An update of the above systematic review, published in 2006, included 24 RCTs that assessed the effectiveness of traction in the management of LBP [4]. The RCTs were selected if they examined any type of traction on acute, subacute, or chronic LBP with or without sciatica. Of the 24 RCTs, only 5 were considered of high quality, and suggested that there was strong evidence that traction was not effective in the management of patients with mixed duration of LBP with or without sciatica. However, there was moderate evidence that autotraction was effective in the management of patients with mixed duration of LBP with or without sciatica.

The most recent update of the above systematic reviews, published in 2013, included 32 RCTs that assessed the effectiveness of traction in management of LBP using the same selection criteria that were used previously [5]. Of the 32 RCTs, 16 studies were considered to have low-risk of bias. The overall conclusion of this systematic review suggested that traction, alone or in combination with other interventions, has little or no impact on the clinical outcomes of pain and function on people with mixed duration of LBP with or without sciatica. However, this systematic review suggested that large, high-quality studies, were still required to make definitive conclusion about traction effectiveness.

Interestingly, rehabilitation providers are reported to be aware of the recommendations from systematic reviews against traction, yet 64% of them disagree with these recommendations and 25% remain undecided [1]. One explanation for this large amount of disagreement may be that rehabilitation providers regularly report the empirical observation that some patients are dramatic responders to lumbar traction [1]. This clinical observation may be driven by the ability of rehabilitation providers to somehow recognize certain clinical patterns that allow them to match patients’ symptomatic presentations to specific traction strategies [2]. This pattern recognition of a traction subgroup has been recommended within the treatment-based classification system, which is commonly utilized by physical therapists [8–10].

Another explanation for this divergence between the continued use of lumbar traction by rehabilitation providers and the recommendations against it from systematic reviews may be related to the variability in the delivery methods of traction among the RCTs included in these systematic reviews [5]. The variability in delivery of traction interventions can stem from using different types of traction, different traction parameters, and different patient populations [5]. When RCTs with different traction methods are pooled together, the overall treatment effect size is diluted.

A number of RCTs suggest that traction can be an effective intervention in the management of patients with LBP. Fritz et al. found that mechanical traction in combination with extension exercises can result in significant improvement in disability and fear-avoidance beliefs after two weeks of treatment compared to extension exercises alone for patients with acute LBP and nerve root compression symptoms [11]. Also, Prasad et al. found that using inversion traction plus physical therapy in patients awaiting surgery for disc herniation helped 77% of them avoid surgery compared to physical therapy alone that helped only 22% avoid surgery [12]. Additionally, Kim et al. found that when prescribing the inversion traction for patients with chronic LBP, the tilt degree of the traction table matters [13]. Kim et al. found the a tilt degree of 60 resulted in improve levels of pain, spine flexibility and trunk extensors strength compared to tilt degrees of 30 or 0 (supine position) [13]. Further, Simmerman et al. found that aquatic traction resulted in significant pain reduction and centralization of symptoms compared to land-based exercises in patients with chronic LBP associated with nerve root compression symptoms [14]. Finally, Diab and Moustafa found that traction in combination with stretching and infrared radiation resulted in significant improvement of pain and disability levels compared to stretching and infrared in patients with chronic LBP at 6 months [15]. Collectively, these individual RCTs point to the potential effectiveness of traction in the treatment of patients with LBP with or without sciatica.

Given the possibility that the treatment effect size could be diluted when heterogeneous studies are pooled together, the purpose of this review is to map the evidence regarding the diversity in traction delivery methods. Specifically, this systematic review will explore the various traction intervention protocols by reporting on traction types, traction parameters, dosage and patients’ characteristics. Assessing the diversity of traction delivery methods will help determine the appropriateness of conducting meta-analysis.

## Methods

This is a systematic review of RCTs that have included some type of lumbar traction as a treatment intervention. The data collection of this systemic review started in September 2016. The first step was to perform a generalized search of the literature using the key words “lumbar traction” in the following databases: Cochrane Central Register of Controlled Trials, MEDLINE, EMBASE, and CINAHL. The search returned several systematic reviews that directly addressed the topic of lumbar traction. The most recent Cochrane systematic review included RCTs of lumbar traction published from inception until August 2012 [5]. From this Cochrane review [5], all of the RCTs contained within the evidence tables were extracted and compared with those found in the evidence tables of the other systematic reviews [3, 7]. This step was necessary to confirm that the recent Cochrane review included all of the RCTs (or more) contained in the evidence tables of the older systematic reviews.

The next step was to update the systematic review by searching for additional RCTs published between September 2012 and December 2016. To identify new RCTs, the following keywords were used to search the same databases: “traction” OR “traction therapy” OR “traction physical therapy” OR “decompression” OR “unloading”; OR “lower back” OR “low back pain” OR lumbar pain OR sciatica OR radiculopathy OR lumbago OR backache. After these additional RCTs were retrieved, two authors (MAlr and MAlm) examined the titles and abstracts to select studies that would potentially be worthy of full text review. After that, the two authors extracted and synthesized the data about the specific traction protocols used in each RCT by reporting on the traction type, traction parameters and patient population. The authors used consensus to agree on which trials would warrant a review of the full text article for potential inclusion in this systematic review. When disagreement occurred, the third author (MS) was consulted to resolve the disagreement.

For a study to be included in this systematic review, it had to be an RCT of patients 18 years of age or older; with acute, subacute or chronic LBP; with or without sciatica. Also, the studies had to include at least one type of traction: manual, auto-traction, gravitational, aquatic and mechanical traction. The traction may or may not have been combined with other interventions such as manual therapy or exercise, with the requirement that traction was the primary intervention. Additionally, any type of comparison group was allowed including placebo, sham or active intervention. Finally, the RCTs must have included at least one clinically relevant outcome measure such as numeric pain scale, self-reported function, global measure of improvement, or return to work. These inclusion criteria are similar to those reported in the most recent Cochrane systematic review [5].

The last step was to create an evidence table that combined the RCTs extracted from the most recent Cochrane systematic review with the new RCTs identified through the updated search (Table 1). Because this review focused on extracting details about the specific traction protocols used in each RCT, there was no need to collect data on quality and risk of bias. This format was used to allow the reader to quickly visualize the similarities and differences in traction protocols across the RCTs.

**Table 1:** Characteristics of randomized trial of lumbar traction

Research parameters	Studies									
	Isner-Horobeti 2016 [17]	Thackeray 2016 [16]	Diab 2013 [15]	Kim 2013 [13]	Prasad 2012 [12]	Simmerman 2011 [14]	Schimmel 2009 [28]	Unlu 2008 [29]	Fritz 2007 [11]	
Symptom duration	Acute and Subacute	Acute, subacute and chronic	Chronic	Chronic	Acute, subacute and chronic	Acute, subacute and chronic	Chronic	Acute	Acute	
Sciatica status	+	+	–	–	+	+	–	+	+	
Traction type	Mechanical	Mechanical	Mechanical	Inversion	Inversion	Aquatic	Mechanical	Mechanical	Mechanical	
Traction rhythm	Continuous	Continuous	Continuous	Intermittent	Intermittent	Continuous	Intermittent	Intermittent	Continuous	
Traction force	50% of body weight	40–60% of body weight	Maximum tolerable force	Unknown	Unknown	2.3 kg ankle weight	50% of body weight	35–50% of body weight	40 to 60% of body weight	
Patient position	Supine	Prone	Supine	Supine	Unknown	Vertical	Supine	Supine	Prone	
Traction frequency	10 sessions, 5 per week, for 2 weeks	12 session for 6 weeks	3 min 1st session, and increased 1 min each subsequent session for a maximum of 20 min. 3 times per week for 10 weeks	Three 3-min inversions, 4 times a week for 8 weeks	Six 2-min inversions, 3 times a week for 4 weeks	1 session for 15 min	25–30 min per session, for 20 sessions for 6 weeks	15 min per session for 5 days a week for 3 weeks	12 min for 12 session for 6 weeks	
Traction combined with other interventions	No	Yes: directional preference exercises	Yes: mixed physical therapy interventions	No	Yes: mixed physical therapy interventions	No	No	No	Yes: directional preference exercises	
Comments	Patient had specific LBP (disc)				Patient had specific LBP (disc)			Patient had specific LBP (disc)		
Research parameters	Studies									
	Harte 2007 [30]	Gudavalli 2006 [31]	Ozturk 2006 [32]	Borman 2003 [33]	Sherri 2001 [20]	Guvenol 2000 [34]	Werners 1999 [35]	Beurskens 1997 [21]	Van der Heijden 1995 [36]	Letchuman 1993 [37]
Symptom duration	Acute and subacute	Chronic	Acute, subacute and chronic	Chronic	Chronic	Subacute and chronic	Acute	Subacute and chronic	Acute, subacute and chronic	Acute, subacute chronic
Sciatica status	+	+/−	+/−	Unknown	+	+	+/−	–	+/−	+/−
Traction type	Mechanical	Manual	Mechanical	Mechanical	Mechanical	Inversion	Mechanical	Mechanical	Mechanical	Mechanical
Traction rhythm	Continuous	Intermittent	Continuous	Unknown	Intermittent	Unknown	Intermittent	Continuous	Continuous	Continuous and intermittent
Traction force	5–60 kg	Unknown	25–50% body weight	Up to 50% of the body weight	50–95 lbs	Unknown	10–20 kg	35–50% of body weight	30 to 50% of body weight maximum	50% of body weight
Patient position	Supine	Prone	Unknown	Supine	Prone	Supine	Supine	Unknown	Unknown	Prone
Traction frequency	10–20 min for 2–3 times a week for 4–6 weeks	2–4 sessions per week for 4 weeks, 9–18 min of traction	15 min per sessions, for 5 sessions per week, for 15 sessions	20 min for 5 sessions per week for 2 weeks	30 min per session for 5 sessions per week for 4 weeks, then once per week for 4 weeks.	5 min on 1st day, 8 min on 2nd day, 10 min on 3rd day and onwards through 7 days (10 days total)	6 sessions for 2–3 weeks	20 min per session, for 12 times over a period of 5 weeks	unknown session duration for 10–12 sessions for 4 consecutive weeks	6 min
Traction combined with other interventions	Yes: mixed physical therapy interventions	No	Yes: modalities	Yes: mixed physical therapy interventions	No	No	No	No	No	No
Comments			Patient had specific LBP (disc)			Patient had specific LBP (disc)		Published data only		
Research parameters	Studies									
	Sweetman 1993 [38]	Tesio 1993 [39]	Konrad 1992 [40]	Ljunggren 1992 [41]	Mathews 1988 [42]	Reust 1988 [43]	Pal 1986 [44]	Ljunggren 1984 [45]	Weber 1983a [19]	
Symptom duration	unknown	subacute and chronic	Subacute	Acute, subacute and chronic	Acute and subacute	Unknown	Acute, subacute and chronic	Subacute	Unknown	
Sciatica status	unknown	+/−	+/−	+	+	+/−	+	+	+	
Traction type	Mechanical	Autotraction	Aquatic	Manual	Autotraction	Mechanical	Mechanical	Autotraction	Manual	
Traction rhythm	Continuous	Intermittent	Unknown	Continuous	Continuous	Continuous	Continuous	Intermittent	Intermittent	
Traction force	1st week 33% body weight, 2nd week 50% body	Unknown	Participant’s weight, gravity plus 3 kg weight on both sides.	300 N	45 kg	5-kg on day 1, 10 kg on day 2, 15 kg on day 3, increasing 5 kg each day up to a maximum of 50 kg	5.5–8.2 kg	Between 33 and 100% of participant’s body weight	40–70 kilopond	
Patient position	Unkonwn	Unknown	Standing	Unknown	Supine or prone	Unknown	Supine	Unknown	Unknown	
Traction frequency	10 min per session, for 3 sessions per week, for unknown number of weeks	3–6 s with 1-min rest for 30–60 min/session. 2–3 times a week for 3–10 sessions.	15 min, 3 times per week, for 4 weeks	10 min per day for 5–7 days	30 min per session for 5 days per week for a maximum of 3 weeks	10 min per day, for 12 sessions, for 12 days	Unknown	Every treatment lasted about 1 h	10–20 s followed by rest for 20 min one time per day	
Traction combined with other interventions	No	No	No	No	No	No	No	No	No	
Comments				Patients had specific LBP (disc).				Patients had specific LBP (disc).	Patients had specific LBP (disc).	
Research parameters	Studies									
	Weber 1983b [19]	Walker 1982 [46]	Coxhead 1981 [47]	Larson 1980 [48]	Bihaug 1978 [49]	Mathews 1975 [50]	Lind 1974 [51]	Weber 1973 [52]	Lidstrom 1970 [53]	
Symptom duration	Unknown	Acute, subacute and chronic	Acute, subacute and chronic	Subacute	Subacute and chronic	subacute and chronic	Acute, subacute and chronic	unknown	Subacute and chronic	
Sciatica status	+	+	+	+	+	+	+/−	+	+	
Traction type	Manual	Mechanical	Mechanical	Autotraction	Autotraction	Mechanical	Autotraction	Mechanical	Mechanical	
Traction rhythm	continuous	Unknown	Intermittent	Unknown	Unknown	Unknown	Unknown	Intermittent	Intermittent	
Traction force	30 kilopond	40–70 kiloponds	unknown	Unknown	70 Kilopond	36.3 kg	Unknown	33% of body weight	Unknown	
Patient position	Unknown	Unknown	Unknown	Prone, supine, sidelying	Prone	Altered at the therapist’s discretion	Unknown	Unknown	Supine	
Traction frequency	10–20 s followed by rest for 20 min one time per day	20 min daily for 4–8 days.	4 weeks	up to 3 treatments within 1 week for less than 1 h	4–12 sessions per week	30 min per day, for 5 days per week, for 3 weeks.	1 h for 1–3 weeks	20 min per session for 5–7 days	Unknown	
Traction combined with other interventions	No	Unknown	No	No	Unknown	No	Yes: bed rest and postural advice.	Unknown	Unknown	
Comments	Patients had specific LBP (disc).		Sciatica was defined as pain as low as the buttock crease							

## Results

**Fig. 1:**
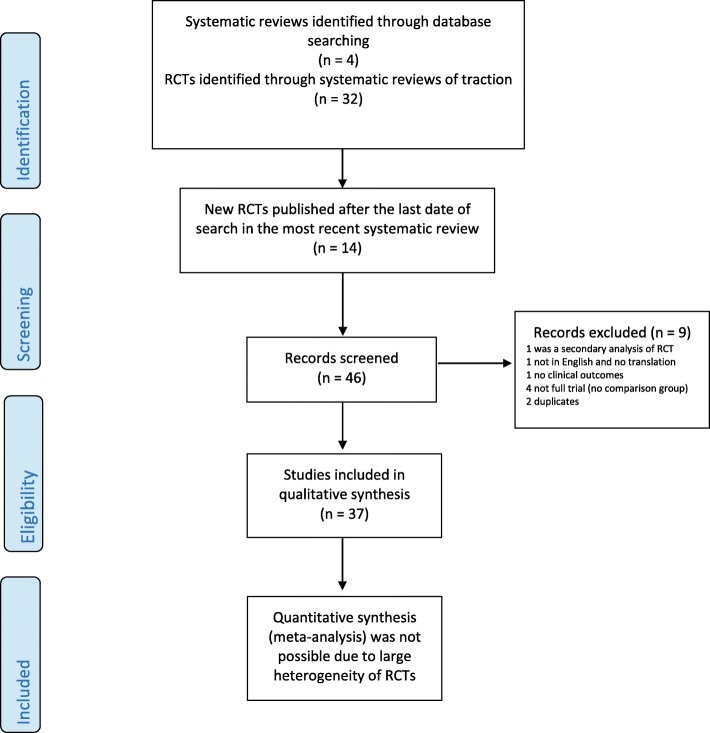
Flow diagram of data searching, screening and inclusion of traction trials. RCT: Randomized controlled trial

The most recent Cochrane systematic review included 32 individual lumbar traction RCTs published from inception through August 2012 [3, 5, 7]. Our updated search identified an additional 14 newer studies that were published between September 2012 and December 2016. Of these newer studies, 5 RCTs were combined in Table 1 with the previously identified 32 RCTs for a grand total of 37 RCTs [12, 13, 15–17]. This search process is summarized in (Fig. 1) [18].

In the columns of Table 1, the primary author and date of each RCT are organized in chronological order. In the rows of Table 1, the qualitative factors and traction parameters of each RCT were included. One study included the results of two RCTs in a single publication [19], so both of those two RCTs were included in Table 1, each reported in a separate column.

Table 2 uses descriptive statistics to summarize categories of traction parameters and patient characteristics from the included RCTs. Of the 37 RCTs, 59.5% used some type of mechanical traction while the remaining studies used auto-traction (16.2%), manual (10.8%), inversion (8.1%), or aquatic traction (5.4%). In 27% of the trials, traction was used in combination with some other type of rehabilitation intervention, such as exercise or physical agents.

**Table 2:** Descriptive statistics of traction and patient characteristics

Traction and patient characteristics	Descriptive statistics *n* (%)
Traction types	
Mechanical	22 (59.5%)
Manual	4 (10.8%)
Auto-traction	6 (16.2%)
Inversion	3 (8.1%)
Aquatic	2 (5.4%)
Traction combined with other intervention	
Traction alone	25 (67.6%)
Modalities	1 (2.7%)
Directional preference	2 (5.4%)
Mixed with physical therapy interventions	5 (13.5%)
Mixed with unknown intervention	4 (10.8%)
Traction force	
Specific amount of weight (2.3–60 kg)	14 (37.8%)
Percentage of body weight (20–100%)	13 (35.1%)
Unknown	10 (27%)
Traction rhythm	
Intermittent	13 (35.1%)
Continuous	14 (37.8%)
Mixed	2 (5.4%)
Unknown	8 (21.6%)
Patient position	
Supine	11 (29.7%)
Prone	6 (16.2%)
Standing or vertical	2 (5.4%)
Mixed	2 (5.4%)
Unknown	16 (43.2%)
Patient symptom duration	
Acute	3 (8.1%)
Subacute	3 (8.1%)
Chronic	6 (16.2%)
Mixed	20 (54.1%)
Unknown	5 (13.5%)
Patient sciatica status	
Present sciatica	22 (59.5%)
Absent sciatica	4 (10.8%)
Mixed	9 (24.3%)
Unknown	2 (5.4%)
Pathology	
Specific low back pain pathology	9 (24.3%)
Non-specific low back pain pathology	28 (75.7%)

The amount of force used during the traction treatment varied widely across these 37 studies, ranging from 2.3 kg in one trial to 100% of body weight in another. In 35% of the RCTs, the amount of traction force was determined by using some arbitrary percentage of the patient’s body weight that varied from 20 to 100%. However, another 37.8% of studies used an arbitrary pre-determined amount of weight ranging anywhere from 2.3 kg to 60 kg as the traction force. The traction rhythm was evenly distributed between continuous and intermittent types of application, with each type of application used 43.2% of the time. In the remaining of the studies, the traction force and rhythm were not clearly described.

The traction session time and treatment frequencies were very difficult to categorize. The traction sessions lasted from 3 to 4 min in duration in some trials, to more than 30 min in other trials. The frequency of application of the traction treatments used in these trials varied from as few as one single session, to as many as 20 traction sessions applied over 6 to 10 weeks.

Other traction parameters were also found to vary widely across these 37 traction RCTs. With respect to patient positioning during the application of traction, 29.7% of the trials had the patient lie in a supine position, 16.2% used prone positioning, 5% applied traction in standing, 3% used a side-lying position, and in 43.2% of the studies there was no clear description of patient positioning.

Regarding the onset and duration of symptoms, 54% of the studies included patients with a mixed acuity of LBP, 16% included only patients with chronic LBP, 8% included only subacute cases of LBP, 8% included only acute cases, and 14% of the studies did not provide any clear description of symptom acuity. With respect to leg symptoms (e.g. sciatica), 59% of the RCTs included only patients with sciatica, 24.3% included a mixture of patients with and without sciatica, 10% included only patients without sciatica, and the remaining studies did not contain any report of leg symptom or sciatica status. In 24% of the included studies, the patients had a specific LBP diagnosis such as herniated disc, while the other 76% of studies did not report any specific pain generator or anatomical cause of the LBP (non-specific LBP).

## Discussion

The results of this systematic review show that there are widespread variations in most of the traction protocols used in the RCTs found in the traction literature. When examining Table 1, each RCT appears to have a distinct combination of traction type and traction parameters applied to different populations of patients with LBP, with or without sciatica. There is no main theme or pattern that emerges about the traction parameters used in the studies included and rated in the previously published systematic reviews. This variability in traction delivery protocols represents a primary gap in the traction literature and might be a key factor that underpins the negative conclusions about traction in the most recent systematic review [5].

### Mechanical and non-mechanical traction

The majority of RCTs that we reviewed used some type of mechanical traction, which involved various devices (e.g. ActiveTrac Table [11], VAX-D decompression [20], SpinaTrac [19], etc.) that used computerized algorithms to produce controlled, intermittent traction forces via motorized pulleys. Although these mechanical devices have the capacity to generate specific forces and rhythms that can be quantified, we found a serious lack of any standardized traction protocol across the RCTs that involved mechanical traction. Instead, the RCTs were found to have great variations in the way the patients were positioned, the duration of traction sessions, the frequency of traction treatment, the amount of traction force applied and the rhythm of traction force. This lack of standardized mechanical traction protocol argues against pooling all of these RCTs under the umbrella term “mechanical traction”, and further casts doubt on any conclusions derived from analyses of the pooled studies through meta-analysis.

There were also some studies that utilized non-mechanical traction interventions, such as manual traction, auto-traction, and gravitational traction. All of these interventions present a challenge when attempting to standardize the treatment because the nature of the used traction force cannot be quantified. Manual traction and auto-traction involve traction forces that are dependent on the skill and strength of the clinician and patient respectively. Gravitational (inversion) traction devices and aquatic traction involve traction forces that are dependent on the suspension effect of gravity or water which varies according to the patient’s body weight and/or externally applied weight attachments. These differences in force levels exist among the patients within any one specific trial or across the RCTs. This suggests that the RCTs utilizing non-mechanical traction interventions should not be pooled together, and any conclusions drawn from these inappropriately pooled studies should be considered circumspect.

### Determining the traction force

In the RCTs that involved mechanical traction devices, the traction forces were applied using either a predetermined amount of weight or a percentage of body weight. In many instances, the predetermined amount of weight was not reported (Tables 1 and 2). We could not find any consistent pattern, explanation or scientific rationale that explained the process by which the specific amount of traction force was determined; rather, the process seemed arbitrary across the RCTs.

Compared to using a predetermined amount of weight, using a percentage of body weight would seem to be a better method to individualize, quantify and standardize the traction force. However, the use of a percentage of body weight also varied widely across the RCTs. Despite the belief that a traction force of 25% of body weight (or above) is reported to create separation between lumbar vertebra [5, 21], it remains to be determined what level or range of traction force is optimal and most therapeutic.

### Duration and frequency of traction session

The application time of traction during each treatment session, and the frequency/total number of treatment sessions also varied and were distinct in almost every RCT, which renders them difficult to categorize. By examining Table 1, these traction parameters of duration and frequency seem to have been chosen arbitrarily, and therefore we could not determine any consistent traction dosage protocol across the RCTs.

### Patients selection for traction

With respect to patients’ characteristics and selection for traction, most of the RCTs included patients with a mixture of symptom duration (i.e. acute, subacute and chronic LBP), with and without sciatica (Table 1). The RCTs did not report any responders and non-responders analyses, which are often performed as a secondary analysis in an attempt to determine if there are any baseline characteristics that might be potential predictors of treatment response. Significant predictor variables can be used to develop clinical prediction rules that can be useful to clinicians for subgrouping patients, and matching treatments with appropriate patient presentations. We only found two such studies; one that developed a preliminary prediction rule for traction and the other that tested this rule within the context of a clinical trial [11, 16]. This suggests that little attention was given to the homogeneity of LBP population that received lumbar traction within RCTs.

When traction (or any treatment) is applied to all patients without regard to subgroup matching, it is not surprising to find mixed results regarding its clinical effectiveness. This situation reminds us of the discordance between clinicians who practice manual therapy and the literature regarding the effectiveness of spinal manipulation for patients with non-specific LBP [22]. Collectively, spinal manipulation studies have been shown to have only modest treatment effect sizes on LBP when the results of those studies are pooled together in systematic reviews [23]. Meanwhile, rehabilitation providers continue to assert its clinical effectiveness, especially for patients who are matched to clinical prediction rules for manipulation [24]. Not surprisingly, when individual manipulation studies applied the concept of subgrouping and administered manipulation in a matched/unmatched design, the clinical effect sizes were much greater [24, 25]. The same principle of subgrouping has been shown to lead to greater treatment effects in trials that matched specific exercises to patients who exhibited a clear directional preference to flexion or extension movements [26, 27].

There is strong face validity to the concept of using spinal traction as a clinical tool for the treatment of LBP. Spinal traction might work by relieving the stress on a painful joint through increasing intervertebral space, loosening adhesions on facet joints and decreasing mechanical stress on the disc [5]. However, there is simply an evidence gap with respect to a validated clinical prediction rule that could guide the selection of traction for those LBP patients who are more likely to be traction responders.

### Future directions for traction

We suggest that future traction research studies should strive toward standardizing the delivery method of traction for patients with LBP. This could be achieved by focusing more on efficacy trials that explore the clinical effects of different dosage parameters including the traction force level, traction rhythm, traction session duration, and traction treatment frequency. It is possible that traction trials have failed to show a treatment effect simply due to suboptimal dosage. Finding the therapeutic dosage level is key for any treatment to succeed.

Also, future traction research should attempt to provide evidence for subgroup(s) of patients as potential traction responders. This would require some modifications to the research design that focus on baseline characteristics of the patient or the clinical examination. For example, RCTs may be improved by considering the patient’s response to a single traction session at first encounter. Patients who show a positive response could be considered to have a “directional preference” to an axial force, and this response could be used as a baseline independent variable in regression models. It would be important to analyze whether the presence of directional preference to an axial force is associated with improved therapeutic effect of traction.

There has been a tendency amongst the RCTs to focus on the inclusion criterion of presence of leg pain (sciatica) and/or signs of nerve root tension, with the assumption that these are important characteristics for a positive response to traction treatment. However, surveys of rehabilitation providers indicate that traction may also be successful with patients who do not present with distal leg symptoms or nerve root compression signs [1, 2]. Future trials should examine if traction force and parameters are different between patients with leg symptoms and patients without leg symptoms.

## Conclusions

RCTs of lumber traction have employed a mixture of traction types, traction parameters and patient populations. The large variability in the delivery of traction intervention provides evidence that the RCTs included in systematic reviews were extremely heterogeneous. This suggests that negative conclusions about the overall clinical effectiveness of lumbar traction should be interpreted with caution. More research about the effectiveness of traction is still necessary, and future trials should consider two important points: (1) discovering optimal dosage and traction parameters to inform the development of standardized traction protocols, and (2) discovering the important baseline variables predictive of successful traction response. By standardizing the traction dosage and parameters, improving patient selection criteria, and response to axial force, more clinically meaningful traction research could be conducted.
